# Neighborhood deprivation, built environment, and overweight in adolescents in the city of Oslo

**DOI:** 10.1186/s12889-023-15261-2

**Published:** 2023-05-03

**Authors:** Sílvia R. Coutinho, Oddbjørn Klomsten Andersen, Nanna Lien, Mekdes K. Gebremariam

**Affiliations:** 1 Department of Nutrition, University of Oslo, Oslo, Norway; 2 Department of Sports Medicine, Norwegian School of Sport Sciences, Oslo, Norway; 3 Department of Community Medicine and Global Health, University of Oslo, Oslo, Norway

**Keywords:** Neighborhood deprivation, Food environment, Physical activity environment, Childhood overweight

## Abstract

**Background:**

Even though the social and built environment characteristics of neighborhoods have been studied as potential determinants of social inequalities in obesity among adults, fewer studies have focused on children. Our first aim was to investigate whether there were differences in the food and physical activity environments between different neighborhood deprivation levels in the city of Oslo. We also explored whether there was an association between the prevalence of overweight (including obesity) among adolescents and (i) neighborhood deprivation levels and (ii) food and physical activity environments of the neighborhoods they live in.

**Methods:**

We conducted a food and physical activity environment mapping (using ArcGIS Pro) in all neighborhoods of Oslo, which were defined by administrative boundaries (sub-districts). The neighborhood deprivation score was calculated based on the percentage of households living in poverty, unemployment in the neighborhood, and residents with low education. A cross-sectional study including 802 seventh graders from 28 primary schools in Oslo residing in 75 out of 97 sub-districts in Oslo was also performed. MANCOVA and partial correlations were ran to compare the built environment distribution between different neighborhood deprivation levels, and multilevel logistic regression analyses were used to explore the effect of neighborhood deprivation and the food and physical activity environments on childhood overweight.

**Results:**

We found that deprived neighborhoods had greater availability of fast food restaurants and fewer indoor recreational facilities compared to low-deprived neighborhoods. Additionally, we observed that the residential neighborhoods of the adolescents with overweight had greater availability of grocery and convenience stores when compared to the residential neighborhoods of the adolescents without overweight. Adolescents living in neighborhoods with high deprivation had a two-fold higher odds (95% CI = 1.1–3.8) to have overweight compared to adolescents living in neighborhoods with low deprivation, regardless of participants’ ethnicity and parental education. However, the built environment did not determine the relationship between neighborhood deprivation and overweight in adolescents.

**Conclusion:**

The neighborhoods in Oslo with higher deprivation levels had more obesogenic characteristics than the low-deprived neighborhoods. Adolescents living in high-deprived neighborhoods were more likely to have overweight than their counterparts from low-deprived neighborhoods. Thus, preventive measures targeting adolescents from high-deprived neighborhoods should be put in place in order to reduce incidence of overweight.

**Supplementary Information:**

The online version contains supplementary material available at 10.1186/s12889-023-15261-2.

## Background

The prevalence of overweight and obesity remains high, despite the significant public health efforts aimed at tackling it. In 2016, the worldwide prevalence of overweight and obesity was 39% in adults and 18% in children [1]. Norway is no exception and the rates of overweight (including obesity) among children continue to be high [2]. In 2017, as reported by The Norwegian Youth Growth Study, 15.8% of 13-year-olds had overweight and obesity [3]. This represents a serious public health problem as obesity during childhood has several adverse effects on growth and pubertal development [4], cardiovascular health harm in the short-term [5], and a well-known association with obesity in adulthood [6].

In addition, the distribution of obesity is not uniform among children. Their socioeconomic contexts appear to be a determinant of how and why obesity manifests itself unequally [7–9], even when adjusting for individual indicators of social status such as family income, parental education, and ethnicity [10–12]. It has been suggested that neighborhoods are potentially relevant contexts, as they hold both social and built environment characteristics that can affect the health of their residents, such as obesity rates [13–17]. A number of systematic reviews have shown that children living in neighborhoods with high rates of poverty and low levels of education have a greater likelihood of experiencing obesity [13, 18, 19].

The way social and physical resources influence the health of neighborhood residents is not limited to a singular socioeconomic determinant [20]. In this regard, neighborhood deprivation scores incorporate constructs composed of multiple indicators of socioeconomic deprivation, such as indicators of neighborhood education, employment, and poverty, among others [21, 22], reflecting the multidimensional socioeconomic aspects of the contexts in which people live [23]. Largely, multidimensional measures of neighborhood deprivation have been positively associated with obesity, i.e., in its broad form, as neighborhood deprivation rises, the risk of obesity increases, irrespective of the socioeconomic individual characteristics of the residents [13, 14, 19, 24].

One of the mechanisms that may explain the pathway between neighborhood deprivation and obesity rates is the increased exposure to the obesogenic environment in socioeconomically disadvantaged neighborhoods [25–27]. The greater exposure to unhealthy high-energy dense food environments and reduced opportunities for physical activity, observed in deprived neighborhoods [27–30], reinforce the argument for the importance of identifying other factors which may also influence the weight-status of the person [13, 15, 17, 31, 32] beyond the individual behavior choices frame.

Even in the reputedly egalitarian Nordic countries, such as Norway, despite its highly regulated welfare system, the higher rates of obesity have often been found in the more socioeconomically deprived groups [33]. The capital Oslo is often referred to as the “divided city” between the east and west sides, where persistent social inequalities are markedly dividing these two areas, with low-income neighborhoods mostly in the east side and the high-income neighborhoods in the west [33]. In the eastern neighborhoods, the percentage of people with lower education is around 73%, while in the western neighborhoods, 36% [34], and the life expectancy of men is 7.5 years lower compared to the western neighborhoods [34]. Factors such as influx of immigrant families, place stigma, and housing market pressure exacerbate the social inequality, and differentiate Oslo from the rest of Norway [35]. Inequalities in the obesity rate among the adult population in Oslo have also been observed, with higher prevalence in the eastern areas compared to the west [36]. However, few studies have been conducted on the association between neighborhood deprivation and obesity among children [9, 37].

In Oslo, similar to elsewhere in Norway, leisure activities and schools are located where the adolescents live. Thus, residential neighborhoods are essential places where youth spend large portions of their time, which suggests that the social and physical environment surrounding might be of particular importance to this group.

Therefore, the aim of this study was first to explore whether there were differences in the food and physical activity environments between neighborhoods of different deprivation levels in Oslo. Furthermore, we also examined whether there was an association between overweight among adolescents and neighborhood deprivation, and neighborhood food and physical activity environments.

## Methods

Data for this study were collected as part of the “Tackling socioeconomic differences in weight development among youth (TACKLE)” project. TACKLE was a project aiming to explore when, how, and why socioeconomic differences in body weight develop during childhood.

### Food and physical activity mapping, and neighborhood deprivation in Oslo’s neighborhoods

#### Neighborhood food and physical activity environments

Administrative boundaries (sub-districts) derived from Oslo Municipality, Agency of City Environment, were used to define neighborhoods. A total of 97 neighborhoods (sub-districts) were included in the analyses. Neighborhood built environment characteristics were analyzed using ArcGIS Pro 2.6.1. (Esri), and availability was measured by counting the number of food outlets or physical activity resources within the area of each administrative boundary.

Data on restaurants, grocery stores, convenience stores, and fast food restaurants were obtained from Prognosesenteret^1^ and Geodata. These food establishments, with the exception of grocery stores, represent the majority of out-of-home eating [38]. The food outlets were classified according to the categories presented in Table S1 (supplementary section), adapted from the works of Glanz et al., 2007 [39], Polsky et al., 2016 [40], and Saelens et al., 2007 [41]. Additionally, the food outlets were grouped as “healthy” food outlets or “unhealthy” food outlets. Grocery stores usually contain diverse options for fruits and vegetables and other healthy options, they are typically considered as healthy food outlets in the literature [42], and have been positively associated with healthy eating in youth [43]. In contrast, convenience stores are usually characterized as supplying high fat, sugary, take-away or snack food, and other unhealthy food options [44], and fast food restaurants are characterized by selling relatively affordable unhealthy energy-dense food options [26]. Regarding all remaining restaurants (i.e. full-service, coffee shops), although the literature shows less consensus around their association with healthy food options, still, research has found that a greater presence of other types of restaurant in relation to the presence of fast food restaurants was associated with a lower probability of developing obesity [40]. Thus, restaurants and grocery stores were grouped as “healthy” food outlets as opposed to the “unhealthy” food outlets, in which convenience stores and fast food restaurants were placed together.

The locations of all food outlets were verified using Google Street View (GSV), and validated in our study [45]. In addition, food outlets not visible from GSV were verified using a national registry of businesses (The Brønnøysund Register Centre). If we were unable to identify the business through either of these sources, the food outlet was deleted from the map.

Data on public green spaces, indoor recreational facilities, small and large outdoors recreational facilities, and public transportation resources were obtained from governmental data sources (Oslo Municipality, Agency for City Environment; Anleggsregisteret, Ministry of Culture and Equality). A detailed list of all the physical activity resources included in the different categories is provided in Table S2 (supplementary section). Satellite imaging from Google and GSV was used to verify the locations of the public green spaces, recreational facilities, and public transportation resources. For small outdoor facilities this was not possible as they were in many cases too small to be detected by satellite imaging or too far away from the street network to be visible from GSV. Similarly, indoor recreational facilities were not visible by satellite imaging or GSV and could thus not be verified.

#### Neighborhood socioeconomic characteristics

Neighborhood sociodemographic data such as population density and minority ethnicity percentage were collected from the Oslo Municipality Statistics Bank [46].

The neighborhood deprivation composite score was constructed adapted from previous works [22, 47, 48]. In this study, the composite score included percentage of households living in poverty, percentage of residents with low education (i.e. people with elementary education or without complete education), both data supplied by the Oslo Municipality Statistics Bank [46], and percentage of unemployment in the neighborhoods, data provided by the Norwegian Institute of Public Health [49]. All data were collected at sub-district level, defined by administrative boundaries, and were from 2020, except percentage of households living in poverty, which was from 2019. The factor weights for the respective measures were calculated based on the equivalent measures presented by the English Index of Multiple Deprivation, a deprivation index with high predictive power in urban areas [22]. The adjusted weights used were household poverty (38.5%), unemployment (38.5%), and low education (23.1%). In this study, high composite scores reflect great neighborhood deprivation. Three levels of neighborhood deprivation were categorized based on the following: low-neighborhood deprivation - below one standard deviation (SD) from the mean; moderate-neighborhood deprivation - within one SD of the mean; high-neighborhood deprivation - above one SD from the mean [48].

### Cross-sectional survey

#### Design and participants

Participants were seventh graders in primary schools in Oslo, participating in the cross-sectional study of the TACKLE project. Based on the Oslo municipality school register, 97 primary schools in Oslo were invited to participate. The schools were screened for eligibility, and schools with few students in the seventh grade and special schools were excluded. A total of 28 schools participated in the study. Due to Covid-19, data were collected from 11 schools in February-March and 17 schools in September-November 2020. All seventh graders (N = 1540) enrolled in the 28 schools were invited to participate. Written informed consent from a parent or legal guardian was obtained for 939 of these students (61%). A total of 897 students (58%) participated in the study. However, fifteen ID-duplicates and two students without ID numbers were excluded from the analysis, leaving a total of 880 participants (57%). A total of 802 participants (52%) had complete data on weight and height, and on neighborhood deprivation, and these constituted the sample for the analyses. The study protocol complies with the Declaration of Helsinki and was approved by The Norwegian Centre for Research Data (NSD).

#### Measures

Data on age, sex, ethnicity, and residence were collected using a self-reported questionnaire. Participant’s ethnicity was assessed using information about the country of birth of both participants and parents. Ethnic minorities were defined as those who had both parents born in a country other than Norway, according to Statistics Norway practice [50]. Parents reported their educational level in the consent form. The parental education variable was divided into three levels: low (no education/has not completed primary school/primary school/lower secondary school/upper secondary school/vocational school (up to two years)); medium (university/college (up to four years)); high (university/college (more than four years)). The highest educated parent determined the parental education level of the family, or else the one available.

To find the neighborhood (sub-district) related to the participant’s residential neighborhood address, a search for each individual address was conducted using an excel spreadsheet containing all addresses in Oslo provided by Oslo Municipality. Residential neighborhood addresses of the participants whose postcodes were missing, incomplete or invalid were removed. A total of 75 out of 97 residential neighborhoods were included in the participants’ sample.

In the first round of data collection (before Covid-19), height was measured to the closest 0.1 cm by using a mobile stadiometer (Seca 217, Hamburg, Germany) while the participants were shoeless. Weight was determined to the nearest 0.1 kg using a digital scale with external display (Seca 899, Hamburg, Germany). The participants were weighed in bare feet and in light clothing. In the second round of data collection (during Covid-19), anthropometric measures were assessed by self-report. The participants were asked to report their height in whole centimeters (cm) and their weight in whole kilos (kg). No differences were found between the participants of the two data collection rounds in any of the sociodemographic and anthropometric characteristics, such as age, sex, ethnicity, parental education, residential neighborhood deprivation levels, and body mass index (BMI) (data not shown). Furthermore, we do not expect these measurement procedures to be correlated with our measures of neighborhood features, since it is implausible that neighborhood conditions might lead to systematic bias in height/weight reporting. Therefore, all participants were analyzed together. Participant’s weight-status, non-overweight vs. overweight and obesity (hereinafter referred to as overweight), was defined by applying the age- and sex- specific International Obesity Task Force (IOTF) BMI (kg/m^2^) cut-offs [51].

To map food and physical activity environments linked to the participants, we followed the same procedure as described above for all neighborhoods in Oslo, but we used individual spatial buffers around participants’ residential neighborhood addresses. We created an 800 m road-network buffer around each participant’s residential neighborhood address. This buffer zone was chosen as it is frequently used in studies with children [52], and it corresponds to approximately a 10–15 min walk [53]. Road-network buffers are preferable over circular buffers as they can account for path barriers such as body of water, busy roads, and train/tram tracks. The road-network was provided by the Norwegian Mapping Authority.

In order to reflect different aspects of participants’ exposure to the built environment, we objectively measured both availability and accessibility to the built environment features. Availability was measured by counting the number of food outlets or physical activity resources within an 800 m road-network buffer around participant’s residential neighborhood address. Accessibility was measured as the distance (in meters) to the closest food outlet or physical activity resource based on the road-network for each participant (within an 800 m buffer).

#### Statistics

Data were first assessed for means and standard deviations to describe the neighborhood characteristics, including the food and physical activity features. Then, multivariate analyses of variance comparing sociodemographic variables between neighborhood deprivation levels, using Bonferroni post hoc test, were used. Since neighborhoods differ in population size and area, we used population density (number of persons/km^2^) as covariate [54]. Thus, multivariate analysis of covariance (MANCOVA) controlling for population density was performed to compare the built environment characteristics between neighborhood deprivation levels. Minority ethnicity percentage was also included as an additional covariate, given the cross-level interactions between neighborhood contextual variables and ethnicity [55]. Pearson correlations and partial correlations were used to test associations between built environment and neighborhood deprivation score, before and after adjusting for the aforementioned covariates.

Descriptive analyses were used to describe the characteristics of participants. To compare the distribution of participants’ sociodemographic characteristics between the non-overweight and overweight groups, student’s t-test was used for the variable age, and Chi-square test was used for the categorical variables. Also, student’s t-tests were used to compare the mean differences in the built environment features of the participants’ residential neighborhoods between the non-overweight and overweight groups. Confounders for the association between neighborhood characteristics and overweight included were individual characteristics such as participant’s ethnicity [56] and parental education [57], multivariate analyses of variance adjusting for those covariates were performed.

Multilevel logistic regression analysis was used to analyze the effect of deprivation levels of the participants’ residential neighborhoods on participants’ overweight. Afterwards, we ran the same analysis controlling for participant’s ethnicity and parental education (Model 2), and then the neighborhood built environment exposures were included (separately for food outlets and physical activity resources, and separate models were built for availability and accessibility (Models 3–8)). The significance was set to 0.05. Data analyses were conducted using the IBM SPSS Statistics for Windows (version 26).

## Results

**Fig. 1:**
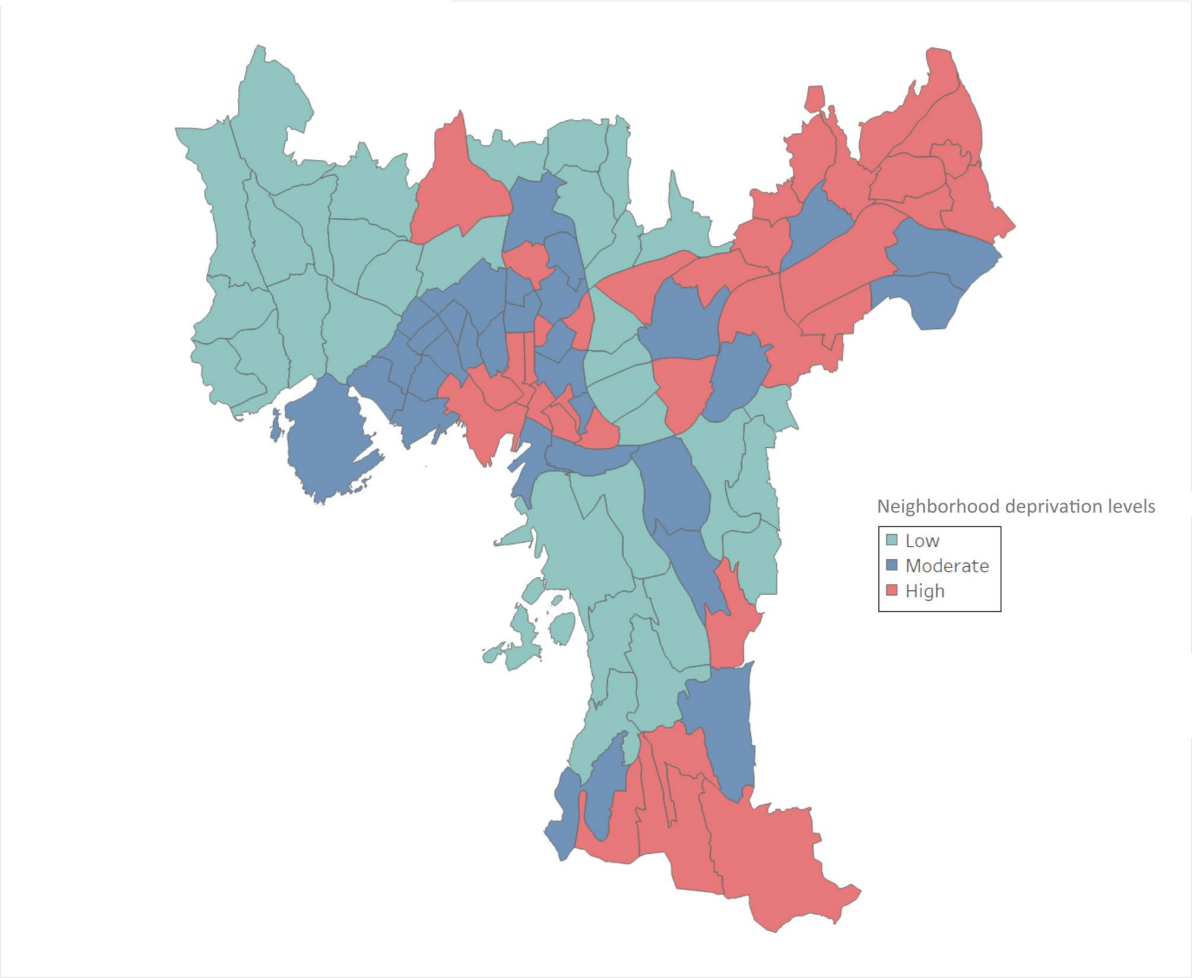
Deprivation levels in Oslo neighborhoods (97 sub-districts)^2^ ^2^Map from Norwegian Mapping Authority and Oslo Municipality Statistics Bank.

Descriptive statistics of the built environment (food and physical activity characteristics), area, and population density of Oslo’s 97 neighborhoods (sub-districts) are presented in Tables S3 and S4 (supplementary section), and the neighborhood deprivation levels across Oslo are shown in Fig. 1.

The maximum number of each type of food outlets per neighborhood were 123 restaurants, 16 grocery stores, 44 convenience stores, and 44 fast food restaurants (Table S3). With regard to availability of physical activity resources, the maximum numbers per neighborhood were 43 total green spaces, about 160 × 10^3^ m^2^ of total area of green spaces, 10 indoor facilities, 31 small outdoor facilities, 28 large outdoor facilities, and up to 50 public transportation stops including bus, train, tram, and metro (Table S4). The neighborhoods’ areas varied between 0.196 km^2^ and 12.175 km^2^, and the total population density between 356 persons/km^2^ and 26,817 persons/km^2^ (both Tables S3 and S4). As pictured in Fig. 1, the neighborhoods with low deprivation levels were mainly distributed on the west side of Oslo, while the neighborhoods with high deprivation levels were predominantly on the east side.

The neighborhoods with moderate or high neighborhood deprivation levels had the highest population density (P < 0.01 and P < 0.05, respectively) and percentage of individuals with a minority ethnic background (P < 0.001, for all) compared to neighborhoods with lower deprivation levels (Table 1). Regarding the built environment features, neighborhoods with high or moderate levels of deprivation had more fast food restaurants (4.9 ± 8.4) and less indoor facilities (1.9 ± 1.8) than the neighborhoods with low deprivation levels (1.3 ± 1.3 and 3.4 ± 2.4, respectively; P < 0.05, for both, Table 1).

**Table 1:** Comparison of sociodemographic and built environment characteristics between neighborhood deprivation levels in Oslo, Norway

	Neighborhood deprivation level^1^ (N = 97)			
	**Low** (34%)	**Moderate** (32%)	**High** (34%)	
**Sociodemographic characteristics**	**Mean (SD)**			**P-value** ^6^
Population density^2^	4202.3 (1840.8)^a, b^	9676.0 (7327.6)^a^	8478.7 (7552.9)^b^	< 0.001
Minority ethnicity (%)	20.0 (5.7)^c, d^	31.0 (7.7)^c, e^	52.0 (13.5)^d, e^	< 0.001
**Availability of food outlets** ^3^	**Mean (SD)**			**P-value** ^7^
• Restaurants	1.8 (2.1)	8.5 (10.1)	10.1 (24.6)	0.08
• Grocery stores	3.4 (2.2)	4.3 (2.4)	3.9 (3.2)	0.46
• Convenience stores	0.9 (1.1)	1.8 (1.5)	2.8 (7.6)	0.24
• Fast food restaurants	1.3 (1.3)^f^	3.7 (3.0)	4.9 (8.4)^f^	0.03
**Availability of PA resources** ^3^	**Mean (SD)**			**P-value** ^7^
• Green spaces	16.2 (9.3)	13.2 (7.9)	16.3 (11.3)	0.26
• Green spaces^4^	21.0 (28.7)	16.5 (17.6)	14.8 (13.3)	0.94
• Indoor facilities	3.4 (2.4)^g^	1.9 (1.8)^g^	2.8 (2.2)	0.04
• Small outdoor facilities	11.9 (6.7)	8.3 (6.2)	11.5 (8.4)	0.08
• Large outdoor facilities	3.2 (5.1)	1.6 (2.4)	2.4 (2.8)	0.27
• Public transportation stops^5^	14.2 (5.0)	11.9 (4.7)	12.5 (9.0)	0.58

^1^Calculated based on low-education, unemployment and poverty percentages in each neighborhood (sub-district); levels grouped into equal-width intervals. ^2^Expressed in population per km^2^. ^3^Number of food outlets/physical activity resources of each type within the total area of each neighborhood (sub-district). ^4^Total area of green spaces (expressed in m^2^ × 10^3^). ^5^Number of public transportation stops including bus, train, tram, and metro. ^6^Multivariate analysis of variance comparing sociodemographic variables between neighborhood deprivation levels, using Bonferroni post hoc test. ^7^Multivariate analysis of covariance (MANCOVA) comparing built environment variables between neighborhood deprivation levels adjusted for population density. Shared letter stands for significant differences between groups: ^b, f, g^P<0.05, ^a^P<0.01, ^c, d, e^P<0.001. SD: standard deviation. PA: physical activity.

Moreover, even after adjusting for population density and ethnicity, positive correlations were observed between neighborhood deprivation scores and the total number of “healthy” (P < 0.05) and “unhealthy” (P < 0.01) food outlets (Table 2). In particular, more availability of restaurants (P < 0.05), convenience stores (P < 0.05), and fast food restaurants (P < 0.01) in the neighborhoods with higher deprivation levels. In addition, the number of public transportation stops was also positively correlated with the neighborhood deprivation score (P < 0.01, Table 2).

**Table 2:** Correlations of food and physical activity environment characteristics with neighborhood deprivation score in Oslo, Norway

Food environment	Neighborhood deprivation score^1^			
	***r*** ^2^	***r*** **partial** ^3^	***r*** **partial** ^4^	***r*** **partial** ^5^
- *Availability of food outlets*:				
• Restaurants	0.168	0.146	0.294**	0.245*
• Grocery stores	0.023	-0.021	0.119	0.012
• Convenience stores	0.202*	0.205*	0.231*	0.249*
• Fast food restaurants	0.265**	0.244*	0.355***	0.304**
*- Availability of food outlets*:				
• “Healthy” food outlets^6^	-0.169	0.128	0.279**	0.221*
• “Unhealthy” food outlets^7^	0.255*	0.233*	0.309**	0.287**
**Physical activity environment**	***r*** ^2^	***r*** **partial** ^3^	***r*** **partial** ^4^	***r*** **partial** ^5^
- *Availability of physical activity resources*:				
• Green spaces	0.137	0.126	-0.019	0.117
• Green spaces^8^	-0.047	0.005	-0.096	0.043
• Indoor facilities	-0.074	-0.056	0.055	0.112
• Small outdoor facilities	0.068	0.123	0.020	0.157
• Large outdoor facilities	-0.056	-0.019	-0.060	0.040
• Public transportation stops^9^	-0.016	0.046	0.090	0.281**

^1^Calculated based on low-education, unemployment and poverty percentages in each neighborhood (sub-district). ^2^*r*: Pearson’s correlation coefficient. ^3^*r* partial: partial correlation coefficient adjusted for population density (population per km^2^). ^4^*r* partial: partial correlation coefficient adjusted for ethnicity (minority ethnicity percentage). ^5^*r* partial: partial correlation coefficient adjusted for population density (population per km^2^) and ethnicity (minority ethnicity percentage). ^6^Merged restaurants + grocery stores, and adjusted for “unhealthy” food outlets. ^7^Merged convenience stores + fast food restaurants, and adjusted for “healthy” food outlets. ^8^Total area of green spaces. ^9^Number of public transportation stops including bus, train, tram, and metro. *P < 0.05, **P < 0.01, ***P < 0.001.

The sociodemographic characteristics of the participants and the levels of deprivation of the participants’ residential neighborhoods are shown in Table 3. The mean age of the respondents was 12.4 ± 0.4 years, just over half were girls (54%), and around 12% had overweight (and 1.1% obesity). About 71% were of Norwegian ethnicity, near 54% of their parents reported high educational attainment, and 59% were living in moderate-deprived neighborhoods.

**Table 3:** Participants’ sociodemographic characteristics and deprivation levels of their residential neighborhoods, overall and divided by weight-status

	Total (N = 802)	Weight-status^1^		
		**Non-overweight** (N = 704)	**Overweight** (N = 98)	
**Participants characteristics**	**Mean (SD)**	**Mean (SD)**		**P-value**
Age (years)	12.4 (0.4)	12.4 (0.3)	12.4 (0.4)	0.24^a^
	**%**	**%**		
Sex (girls)	54.0	54.8	52.0	0.60^b^
Weight-status:				
• Non-overweight	87.8	---	---	
• Overweight	12.2	---	---	
Ethnicity:				< 0.001^b^
• Norwegian	71.3	74.9	45.3	
• Minority	28.7	25.1	54.7	
Parental education^2^:				< 0.001^b^
• High	53.7	56.5	33.7	
• Medium	22.0	21.7	24.2	
• Low	24.3	21.7	42.1	
**Neighborhood deprivation level** ^3^				< 0.001^b^
• Low	21.3	23.5	6.2	
• Moderate	59.4	58.5	64.9	
• High	19.4	18.0	28.9	

^1^IOTF body mass index cut-offs adjusted for child age and sex. ^2^Low: from no education to vocational school; medium: university/college up to four years; high: university/college more than four years. ^3^Calculated based on low-education, unemployment and poverty percentages in each participants’ residential neighborhoods (75 in total); levels categorized as described in the methods section. ^a^Comparison of mean age between non-overweight and overweight groups (student’s t-test). ^b^Chi-square test comparing the distribution of participants’ sociodemographic characteristics and deprivation levels of the participants’ residential neighborhoods between non-overweight and overweight groups. SD: standard deviation.

The proportion of adolescents with overweight was significantly larger in the neighborhoods with high deprivation levels (28.9%) than in the low-deprived neighborhoods (6.2%; P < 0.001, Table 3). Moreover, adolescents living in neighborhoods with high deprivation levels were two times (95% CI = 1.1–3.8) more likely to have overweight compared to those living in neighborhoods with low deprivation levels, after adjusting for ethnicity and parental education (Table 4).

**Table 4:** Multivariate logistic models, adjusted for school-clustering, to analyze neighborhood deprivation levels’ effect on participant’s overweight^1^

		OR (95% CI)
Model 1	Neighborhood deprivation level (Ref. Low)	
	Moderate	2.99 (1.51–5.88)
	High	3.81 (1.96–7.43)
Model 2	Neighborhood deprivation level (Ref. Low)	
	Moderate	1.86 (0.95–3.64)
	High	2.03 (1.08–3.82)
	Ethnicity (Ref. Norwegian ethnicity)	
	Minority	2.25 (1.33–3.82)
	Parental education (Ref. High)	
	Medium	0.89 (0.48–1.66)
	Low	0.58 (0.32–1.05)
Model 3^a^	Neighborhood deprivation level (Ref. Low)	
	Moderate	1.71 (0.88–3.33)
	High	1.86 (0.94–3.65)
- *Availability of food outlets*^2^:	• Restaurants	1.01 (0.97–1.05)
	• Grocery stores	1.05 (0.95–1.15)
	• Convenience stores	1.04 (0.97–1.12)
	• Fast food restaurants	0.96 (0.84–1.09)
Model 4^a^	Neighborhood deprivation level (Ref. Low)	
	Moderate	1.80 (0.92–3.55)
	High	1.84 (0.96–3.53)
- *Availability of food outlets*^2^:	• “Healthy” food outlets^3^	1.00 (0.97–1.02)
	• “Unhealthy” food outlets^4^	1.03 (0.96–1.09)
Model 5^a^	Neighborhood deprivation level (Ref. Low)	
	Moderate	1.90 (0.96–3.75)
	High	1.90 (1.00–3.60)
- *Accessibility of food outlets*^5^:	• Restaurants	0.97 (0.93–1.02)
	• Grocery stores	1.01 (0.91–1.11)
	• Convenience stores	0.99 (0.94–1.04)
	• Fast food restaurants	1.00 (0.93–1.08)
Model 6^a^	Neighborhood deprivation level (Ref. Low)	
	Moderate	1.88 (0.96–3.71)
	High	1.89 (0.99–3.58)
- *Accessibility of food outlets*^5^:	• “Healthy” food outlets^3^	0.99 (0.95–1.02)
	• “Unhealthy” food outlets^4^	1.00 (0.96–1.03)
Model 7^a^	Neighborhood deprivation level (Ref. Low)	
	Moderate	1.88 (0.95–3.75)
	High	2.00 (1.01–3.98)
- *Availability of PA resources*^2^:	• Green spaces	1.00 (0.97–1.04)
	• Green spaces^6^	0.98 (0.94–1.03)
	• Indoor facilities	1.09 (0.98–1.22)
	• Small outdoor facilities	0.98 (0.94–1.03)
	• Large outdoor facilities	1.01 (0.88–1.15)
	• Public transportation stops^7^	1.01 (0.97–1.06)
Model 8^a^	Neighborhood deprivation level (Ref. Low)	
	Moderate	1.83 (0.93–3.63)
	High	2.09 (1.09–3.99)
- *Accessibility of PA resources*^5^:	• Green spaces	1.02 (0.91–1.13)
	• Indoor facilities	0.94 (0.88–1.01)
	• Small outdoor facilities	0.99 (0.89–1.10)
	• Large outdoor facilities	1.04 (0.98–1.10)
	• Public transportation stop^8^	1.07 (0.96–1.19)

^1^IOTF body mass index cut-offs adjusted for child age and sex. ^2^Number of food outlets/physical activity resources of each type within an 800 m radius buffer around the residential neighborhood address of the participant. ^3^Merged restaurants + grocery stores. ^4^Merged convenience stores + fast food restaurants. ^5^Shortest distance in meters from the residential neighborhood address of the participant to the nearest food outlet/physical activity resource of each type (within an 800 m radius buffer). ^6^Total area of green spaces. ^7^Number of public transportation stops within an 800 m radius buffer around the residential neighborhood address of the participant, including bus, train, tram, and metro. ^8^Nearest public transportation stop, including bus, train, tram, and metro, within an 800 m radius buffer around the residential neighborhood address of the participant. ^a^Model adjusted as well for the ethnicity of the participants and parental education. OR: odds ratio. CI: confidence interval. PA: physical activity

Concerning the built environment of the participants’ residential neighborhoods, there were significant differences between the groups of adolescents with and without overweight in the food environment outcomes only (Table 5). Particularly, restaurants (P = 0.02), grocery (P = 0.01) and convenience stores (P = 0.01) appeared to be more available in the residential neighborhoods of the adolescents with overweight than in the neighborhoods of the adolescents without overweight. Additionally, there was a trend (P = 0.06) towards greater availability of fast food restaurants in the residential neighborhoods of the adolescents with overweight (4.0 ± 4.9) than in the residential neighborhoods of the adolescents without overweight (2.9 ± 4.5). In relation to food outlets accessibility, in the group of adolescents with overweight, both restaurants (P = 0.03) and grocery stores (P = 0.02) were closer in their residential neighborhoods than in the residential neighborhoods of the adolescents without overweight. However, after controlling for participants’ ethnicity and parental education, availability of restaurants and accessibility to grocery stores were no longer significantly different between the respective residential neighborhoods of the adolescents with or without overweight (Table 5). Still, independently of participants’ ethnicity and parental education, the residential neighborhoods of the adolescents with overweight presented greater availability of both “healthy” (mainly grocery stores, P < 0.05) and “unhealthy” (mainly convenience stores, P < 0.05) food outlets, and a tendency (P = 0.06) towards greater accessibility to “healthy” food outlets (mainly restaurants, P = 0.06) when compared to the residential neighborhoods of the adolescents without overweight (Table 5). Nevertheless, none of the characteristics of the built environment studied showed to be a significant determinant of the relationship between neighborhood deprivation and overweight in adolescents (Table 4).

**Table 5:** Built environment characteristics in the residential neighborhoods of the participants, overall and divided by weight-status

	Total (N = 802)	Weight-status^1^					
**Food environment**		**Non-overweight** (N = 704)		**Overweight ** (N = 98)			
- *Availability of food outlets*^2^:	**Mean (SD)**	**Mean (SD)**			**P-value** ^9^	**P-value** ^10^	**P-value** ^11^
• Restaurants	5.4 (13.5)	5.1 (13.2)		8.1 (14.4)	0.02	0.07	0.06
• Grocery stores	3.7 (3.9)	3.6 (3.8)		4.6 (4.4)	0.01	0.03	0.04
• Convenience stores	1.4 (2.8)	1.3 (2.8)		1.9 (3.1)	0.01	0.05	0.03
• Fast food restaurants	3.0 (4.7)	2.9 (4.5)		4.0 (4.9)	0.06	0.06	0.09
• “Healthy” food outlets^3^	9.1 (16.7)	8.6 (16.2)		12.7 (18.1)	0.01	0.05	0.05
• “Unhealthy” food outlets^4^	4.4 (6.4)	4.2 (6.2)		5.9 (7.0)	0.02	0.02	0.03
- *Accessibility of food outlets*^5^:	**Mean (SD)**	**Mean (SD)**			**P-value** ^9^	**P-value** ^10^	**P-value** ^11^
• Restaurants	921 (737)	939 (759)		751 (629)	0.03	0.06	0.04
• Grocery stores	524 (319)	541 (329)		457 (267)	0.02	0.19	0.06
• Convenience stores	995 (593)	1001 (590)		905 (583)	0.79	0.16	0.14
• Fast food restaurants	723 (440)	736 (446)		640 (384)	0.29	0.15	0.11
• “Healthy” food outlets^3^	1445 (943)	1480 (976)		1208 (771)	0.05	0.06	0.03
• “Unhealthy” food outlets^4^	1719 (901)	1737 (903)		1545 (831)	0.76	0.10	0.08
**Physical activity environment**							
- *Availability of PA resources*^2^:	**Mean (SD)**	**Mean (SD)**			**P-value** ^9^	**P-value** ^10^	**P-value** ^11^
• Green spaces	13.6 (8.6)	13.5 (8.6)		14.4 (9.1)	0.61	0.61	0.30
• Green spaces^6^	9.9 (6.2)	9.8 (6.1)		10.0 (6.6)	0.14	0.99	0.71
• Indoor facilities	2.2 (2.2)	2.1 (2.2)		2.5 (2.4)	0.35	0.13	0.10
• Small outdoor facilities	9.9 (7.0)	9.9 (7.1)		9.9 (6.6)	0.16	0.94	0.86
• Large outdoor facilities	1.8 (2.1)	1.8 (2.2)		1.8 (2.1)	0.82	0.86	0.88
• Public transportation stops^7^	8.3 (5.5)	8.2 (5.4)		9.2 (6.1)	0.30	0.09	0.25
- *Accessibility of PA resources*^5^:	**Mean (SD)**	**Mean (SD)**			**P-value** ^9^	**P-value** ^10^	**P-value** ^11^
• Green spaces	352 (209)	355 (210)	358 (223)		0.14	0.97	1.00
• Indoor facilities	696 (369)	707 (374)	661 (355)		0.82	0.27	0.21
• Small outdoor facilities	437 (261)	449 (264)	406 (236)		0.24	0.48	0.28
• Large outdoor facilities	886 (445)	887 (444)	904 (470)		0.62	0.55	0.59
• Public transportation stop^8^	361 (215)	362 (219)	361 (201)		0.45	0.47	0.44

^1^IOTF body mass index cut-offs adjusted for child age and sex. ^2^Number of food outlets/physical activity resources of each type within an 800 m radius buffer around the residential neighborhood address of the participant. ^3^Merged restaurants + grocery stores. ^4^Merged convenience stores + fast food restaurants. ^5^Shortest distance in meters from the residential neighborhood address of the participant to the nearest food outlet/physical activity resource of each type (within an 800 m radius buffer). ^6^Total area of green spaces (expressed in m^2^ × 10^3^). ^7^Number of public transportation stops within an 800 m radius buffer around the residential neighborhood address of the participant, including bus, train, tram, and metro. ^8^Nearest public transportation stop, including bus, train, tram, and metro, within an 800 m radius buffer around the residential neighborhood address of the participant. ^9^Differences between non-overweight and overweight groups in the availability and accessibility of food outlets/physical activity resources in the participants’ residential neighborhoods (student’s t-test). ^10^Differences between non-overweight and overweight groups in the availability and accessibility of food outlets/physical activity resources adjusted for the ethnicity of the participants (MANCOVA). ^11^Differences between non-overweight and overweight groups in the availability and accessibility of food outlets/physical activity resources adjusted for parental education (MANCOVA). SD: standard deviation. PA: physical activity

## Discussion

In this study, we found that neighborhoods in Oslo have different obesogenic environments depending on their deprivation levels. In particular, deprived neighborhoods had greater availability of fast food restaurants and lower availability of indoor facilities compared to neighborhoods with low deprivation levels. In addition, we found in our sample of participants that adolescents from deprived neighborhoods were more likely to have overweight than adolescents from advantaged neighborhoods (low-deprived neighborhoods), even when accounting for individual-level sociodemographic characteristics, such as ethnicity and parental education. However, the association between neighborhood deprivation and overweight was not explained by the differences in the food and physical activity environments.

Living in deprived neighborhoods has been implied to increase the risk of unhealthy behaviors as a result of higher exposure to fast food restaurants [26, 58] and limited access to grocery stores [59–61]. Our results support that high-deprived neighborhoods had more fast food restaurants than the low-deprived neighborhoods. However, we did not find significant differences in the number of grocery stores among the three neighborhood deprivation levels, which was not in line with the findings of previous studies that found greater availability of grocery stores in low-deprived neighborhoods [59–61]. Variations in the types of grocery stores evaluated, neighborhood definitions, sample sizes, and the ecological study designs may explain the different results [62].

When food outlets were grouped by “healthy” and “unhealthy” food outlets, the availability of both groups was greater as the neighborhood deprivation score increased. This is partially in agreement with previous systematic reviews, indicating that deprived neighborhoods have greater availability of “unhealthy” food outlets, but generally a lower availability of “healthy” food outlets in such neighborhoods [16, 25, 28, 29]. In our findings, the greater availability of “healthy” food outlets in the more deprived neighborhoods was mostly due to the higher presence of restaurants in those neighborhoods. However, we cannot assure that all restaurants across the neighborhoods were promoting the same food environment, and thus equally exposed the residents to healthy food choices. As evidenced by Saelens et al. (2007), the environment consumers’ experience within restaurants, even within the same type, varies considerably among restaurants (e.g., prices, food promotions, number of healthy food choices, etc.), and, hence, can rearrange individuals’ food choices patterns [41]. Additionally, a recent study on obesogenic food environment and youth found that in socioeconomically deprived neighborhoods, full-service restaurants had greater promotion of unhealthy food options for their residents compared to restaurants in low-deprived neighborhoods [63], a previous paper had already observed the same pattern that unhealthy food options were strongly promoted in restaurants in deprived neighborhoods [64].

On the other hand, the greater availability of “unhealthy” food outlets in higher-deprived neighborhoods seen in this study was due to the greater number of fast food restaurants and convenience stores in these neighborhoods. This is consistent with studies from Canada and England showing a linear increase between neighborhood deprivation and the number of fast food outlets [26, 58], and with more convenience stores in the deprived neighborhoods found in other studies [59, 65]. The implications of these characteristics of the food environment in the deprived neighborhoods, i.e., high exposure to fast food, which is not counterbalanced by higher exposure to grocery stores, are that residents from these neighborhoods can have more access to energy dense, nutrient-low, and highly processed food products.

Regarding the differences in the physical activity environment, in the current study a lower availability of indoor facilities was seen in the more deprived neighborhoods. The relation between neighborhood deprivation and the availability of recreational facilities in the neighborhoods has been demonstrated in some studies, but with contradictory findings [25, 66]. In particular, some studies have outlined that higher deprived neighborhoods have fewer physical activity resources (including both indoor and outdoor facilities) than neighborhoods with lower deprivation levels [27, 67]. Whereas, other studies reported no differences [68, 69], or even better access to both indoor and outdoor recreational facilities in high-deprived neighborhoods [66], or, similar to our findings, only inequalities in terms of the availability of indoor recreational facilities, with fewer opportunities for physical activity among the residents of deprived neighborhoods [70].

Conversely, we observed higher prevalence of public transportation stops in more deprived neighborhoods, contradicting previous research that found neighborhood deprivation to be linked with transport disadvantage [71]. However, in our study, this observation may just reflect the urbanization plan connected to densely populated inner areas, regardless of the deprivation levels of the neighborhoods [47].

Overall, these inconsistent results may be related to the specificities of different urban designs, land use patterns, and neighborhood deprivation measurements (separate indicators versus composite scores) used in each study [72]. In addition, despite the availability of physical activity resources being an important measure of the built environment, combined measures involving equally quantitative and qualitative aspects of the neighborhood physical activity environment can provide a more rigorous picture of the reality of deprivation neighborhoods contexts. Namely, neighborhood features perceived by the residents, like aesthetic appeal and safety, may be as relevant as availability, given the positive associations documented between these qualitative measures of the neighborhoods and the physical activity levels of their residents in low-deprived neighborhoods [73].

In our sample of participants, 29% of the adolescents living in high-deprived neighborhoods had overweight, compared to only 6% in the low-deprived neighborhoods. Moreover, in this study, adolescents living in high-deprived neighborhoods were two times more likely to have overweight than adolescents from low-deprived neighborhoods, even when adjusting for ethnicity and parental education. This finding corroborates several other studies that have reported that overweight in childhood affects unequally the more socioeconomically deprived areas, where children have a high BMI and an increased risk of obesity [13, 18, 19].

Previous evidence has suggested that residents from deprived neighborhoods tend to have a higher BMI where their neighborhoods are characterized by relative prevalence of fast food outlets and constraints on physical activity resources [17, 28, 30]. In the present study, when the neighborhood built environment was compared between the two BMI groups (non-overweight vs. overweight), we only found differences in the food environment characteristics, where there was greater availability of both grocery and convenience stores in the neighborhoods of the adolescents with overweight than in the neighborhoods of the adolescents without overweight. Further, in this study, the built environment features studied did not seem to drive the relationship between neighborhood deprivation and overweight in adolescents.

This is not to say that food and physical activity environments are not important for children’s development, given the increasing influence that environmental characteristics have as children age and become more independent from their families in their interactions with the built environment [72]. Accordingly, maybe the adolescents of our sample were not yet old enough to be influenced by the food and physical activity resources in their neighborhoods. In this regard, this suggests that perhaps other factors that we were unable to adjust for, such as parents‘ lifestyle, household income, psychosocial resources to protect their children from obesogenic environmental exposures [74, 75], and/or differences in perceived social norms between adolescents from different socioeconomic neighborhoods [37], could have acted as mediators between the built environment and adolescents’ weight-status [76, 77]. Longitudinal studies with repeated measurements of neighborhood socioeconomic conditions, built environment, and childhood overweight are needed to disentangle these complex causal relationships.

Nevertheless, if nothing changes in the neighborhood context of these adolescents, growing up in more deprived neighborhoods, and thus being exposed in the long-term to a more favorable obesogenic environment, the risk of obesity incidence in early adulthood is increased [78].

In addition, the residents of deprived neighborhoods may also be deprived of their social and political influence to demand for better and protective live conditions, and to oppose for unwanted unhealthy built environments. Municipalities and decision-makers, who can influence the number and types of goods and resources allocated, in favor of these populations, are therefore essential to tailor the neighborhood built environment. For example, governmental decisions such as zoning laws that can limit the amount of fast food outlets in the high-deprived neighborhoods, along with investments to develop recreational facilities for adolescents, involving community participation in identifying relevant barriers and facilitators that may influence their children’s physical activity opportunities in the high-deprived neighborhoods.

This study has several strengths. We considered a wide range of built environment characteristics, including a variety of both food outlets and physical activity resources, in contrast to most studies [15]. Additionally, the built environment variables were objectively measured using ArcGIS Pro software. Besides, we were able to capture different nuances of the adolescents’ built environment context, by including both availability and accessibility measures. For instance, the presence of one fast food restaurant in the neighborhood could reflect poor availability. However, it could still represent high proximity if this fast food restaurant was close to the participant’s residential address. Finally, although the neighborhood deprivation construct used in this study did not exhaust the domain of all socioeconomic indicators, a composite neighborhood deprivation score comprising three sociodemographic parameters was used to assess the neighborhood deprivation levels, as opposed to use a single measure representing neighborhood income or employment distributions or educational compositions. However, this study also has some limitations. Similar to many studies in the field, a cross-sectional design was used; hence, it has a limited understanding about the relationship between neighborhoods and overweight in adolescents, and little can be said about how youth interact with their neighborhoods. Also, we did not account for additional features to assess the built environment, such as price or nutritional value of food products, within each food outlet, or alternatives to food shopping, such as home food delivery, or prices connected to gyms or other recreational facilities, or perceived neighborhood features, like aesthetic appeal and safety. These factors may be more important to consider in understanding the relationship between overweight and the built environment than simply counting food outlets or physical activity resources. Moreover, while street network buffers compared to census tracts are considered a more accurate representation of a person’s neighborhood, they must still be considered artificial neighborhoods, given they may not reflect the actual space children use, which in turn could underestimate the impact of the built environment [79]. Furthermore, we relied on secondary data sources to collect the built environment features. These databases are rarely perfect, and limit our ability to ensure the rigidity of the data collection and the completeness of the data. Nevertheless, with the exception of small outdoor and indoor recreational facilities, we were able to validate the existence of the environmental features through GSV or satellite imaging, which can be considered a strength [80]. Further, although some sociodemographic variables were included as covariates in the analyses, potential biases associated with neighborhood self-selection cannot be dismissed. Namely, in our analyses indicators of socioeconomic position such as household income were not accounted for. Still, even though each indicator of socioeconomic position has a unique contribution in capturing aspects of socioeconomic context, parental education has been shown to have the greatest influence on children’s health [18, 81]. Furthermore, due to Covid-19 restrictions, one of the rounds of anthropometric measurements was evaluated by self-report, and although reported weight/height data are a reasonably valid alternative to measure children’s BMI [82], errors of measurement cannot be excluded. Because this was an exploratory study, and data concerning rates of childhood overweight at the neighborhood level were not included, no conclusions concerning the actual impact of neighborhood built environment on overweight can be outlined. Finally, no adjustments were made for multiple comparisons, though several multilevel regression models were performed. However, it has been argued that for large samples involving objective observations, adjustments for multiple comparisons are not always required [83].

## Conclusion

Our study results indicated the same pattern of socioeconomic inequalities in overweight as other high-income countries, namely a higher proportion of childhood overweight in the more deprived neighborhoods. However, the association between neighborhood deprivation and overweight was not explained by the differences in the food and physical activity environments. Nevertheless, neighborhood deprivation in Oslo was characterized by a more obesogenic environment, i.e. the deprived neighborhoods had a higher density of fast food outlets and less indoor recreational facilities than the socioeconomic advantaged neighborhoods. Longitudinal studies that explore neighborhood deprivation and the incidence of childhood obesity before and after environmental changes such as lower availability of fast food outlets and creation of recreational facilities are needed to strength the inferences regarding causal effects of the social and built environment characteristics of the neighborhoods.

## Electronic supplementary material

Below is the link to the electronic supplementary material.

Supplementary Material 1

Supplementary Material 2

Supplementary Material 3 and 4

## Data Availability

The datasets used and/or analyzed during the current study are available from the corresponding author upon reasonable request.

## Footnotes

1 It is an independent market analysis company that specializes in the Nordic construction and property markets. The company is employee-owned and has no other owner-interest in the industry, which ensures the impartiality of their analyses.
