# Supplementary material for: Neighborhood deprivation, built environment, and overweight in adolescents in the city of Oslo
Source: BMC Public Health. 2023 May 3;23:812. doi: 10.1186/s12889-023-15261-2 (PMC10155174; doi:10.1186/s12889-023-15261-2)
Supplement: Supplementary file 1 — Supplementary Material 1 [file 12889_2023_15261_MOESM1_ESM.docx]

Table S1. Classification of the food outlets^1^

| **Food outlet** | **Definition** |
| --- | --- |
| Restaurants | Restaurants were classified as either full-service (meals are generally served *a-la-carte* and eaten on the premises) or other restaurants such as cafes or coffee shops |
| Grocery stores | Store that primarily retails a general range of food products, which may be fresh or packaged |
| Convenience stores | Small retail food store, with extended opening hours, that stocks food products that generally includes snacks and/or a limited range of household goods |
| Fast food restaurants | Fast food chains and locally owned fast food restaurants with a minimal service. Provision of standardized foods and/or partially prepared foods (mainly deep-fried foods) that are supplied few minutes after ordering |

^1^Adapted from the works of Glanz et al., 2007 (1), Polsky et al., 2016 (2), and Saelens et al., 2007 (3).

**References**

1. Glanz K, Sallis JF, Saelens BE, Frank LD. Nutrition Environment Measures Survey in stores (NEMS-S): development and evaluation. American journal of preventive medicine. 2007;32(4):282-9.

2. Polsky JY, Moineddin R, Dunn JR, Glazier RH, Booth GL. Absolute and relative densities of fast-food versus other restaurants in relation to weight status: Does restaurant mix matter? Preventive medicine. 2016;82:28-34.

3. Saelens BE, Glanz K, Sallis JF, Frank LD. Nutrition Environment Measures Study in restaurants (NEMS-R): development and evaluation. American journal of preventive medicine. 2007;32(4):273-81.
