# Supplementary material for: Neighborhood deprivation, built environment, and overweight in adolescents in the city of Oslo
Source: BMC Public Health. 2023 May 3;23:812. doi: 10.1186/s12889-023-15261-2 (PMC10155174; doi:10.1186/s12889-023-15261-2)
Supplement: Supplementary file 2 — Supplementary Material 2 [file 12889_2023_15261_MOESM2_ESM.docx]

Table S2. Type of recreational facilities

| **Indoor facilities** |
| --- |
| Multi-functional sports halls |
| Indoor swimming facilities |
| Indoor climbing facilities |
| **Small outdoor facilities** |
| Tennis courts |
| Small ball courts |
| Hockey fields |
| BMX and skateboard facilities |
| Track & field facilities |
| Small ski and alpine facilities |
| Climbing walls |
| Parkour facilities |
| Swimming facilities |
| **Large outdoor facilities** |
| Soccer fields |
| Cricket fields |
| **Parks and green space** |
| Maintained parks (class A-C)^1^ |
| Unmaintained parks (class D)^1^ |
| **Public transportation** |
| Bus stop |
| Train stop |
| Tram stop |
| Metro stop |

^1^Oslo municipality categorizes parks in four categories, where category A-C = maintained parks with or without benches, toilets and/or fountains and category D = unmaintained green space and forest areas with maintained paths.

**Categorization of facilities**

Indoor recreational facilities were mainly made up of multi-functional sports halls. Outdoor recreational facilities were disaggregated into small and large outdoor recreational facilities. Oslo municipality has its own categorization of smaller outdoor recreational facilities, which includes a vast range of facilities (table tennis, smaller courts/fields, obstacle courses, basketball courts, etc.). We chose to retain this categorization, but we also included track and field facilities, ice hockey fields, BMX facilities, skateboard facilities, and ski facilities in this category. Although ice hockey fields and ski facilities have the potential to be large facilities, mostly small facilities of this category exist within the city boarders. Large recreational facilities were almost exclusively made up of soccer fields. Large recreational facilities and parks were presented as polygon layers to better reflect the rather large land area they take up, while the remaining recreational facilities were presented as point layer.
