# Supplementary material for: Neighborhood deprivation, built environment, and overweight in adolescents in the city of Oslo
Source: BMC Public Health. 2023 May 3;23:812. doi: 10.1186/s12889-023-15261-2 (PMC10155174; doi:10.1186/s12889-023-15261-2)
Supplement: Supplementary file 3 — Supplementary Material 3 and 4 [file 12889_2023_15261_MOESM3_ESM.docx]

Table S3. Characteristics of the food environment in Oslo neighborhoods

| **Neighborhood** (sub-districts) | **Number of restaurants** | **Number of grocery stores** | **Number of convenience stores** | **Number of fast food restaurants** | **Area** (km^2^) | **Population density** (population per km^2^) |
| --- | --- | --- | --- | --- | --- | --- |
| Abildsø | 0 | 0 | 0 | 1 | 1.668 | 2294 |
| Ammerud | 0 | 1 | 1 | 1 | 1.315 | 5839 |
| Bekkelaget | 0 | 3 | 1 | 3 | 12.175 | 814 |
| Bislett | 13 | 5 | 1 | 4 | 0.428 | 22348 |
| Bispevika | 24 | 4 | 1 | 1 | 10.240 | 356 |
| Bjølsen | 6 | 7 | 0 | 1 | 0.693 | 10850 |
| Bjørndal | 0 | 3 | 0 | 1 | 6.289 | 1437 |
| Bjørnerud | 0 | 0 | 0 | 0 | 1.489 | 4708 |
| Bygdøy | 3 | 2 | 0 | 0 | 8.385 | 450 |
| Bøler | 0 | 3 | 1 | 2 | 1.551 | 5444 |
| Disen | 0 | 1 | 0 | 0 | 1.040 | 4572 |
| Dælenenga | 6 | 2 | 0 | 0 | 0.196 | 22918 |
| Ellingsrud | 0 | 4 | 2 | 0 | 1.594 | 4698 |
| Enerhaugen | 6 | 4 | 1 | 6 | 0.398 | 19018 |
| Ensjø | 3 | 7 | 1 | 5 | 1.250 | 7724 |
| Etterstad | 1 | 4 | 0 | 0 | 0.931 | 4685 |
| Fagerborg | 5 | 4 | 2 | 1 | 0.339 | 15454 |
| Fossum | 3 | 3 | 2 | 7 | 0.431 | 12084 |
| Frogner | 5 | 1 | 1 | 1 | 1.158 | 5983 |
| Frognerparken | 8 | 3 | 1 | 1 | 0.660 | 10256 |
| Furuset | 1 | 2 | 1 | 3 | 3.510 | 2898 |
| Godlia | 3 | 2 | 3 | 1 | 2.717 | 3265 |
| Grefsen | 3 | 3 | 1 | 1 | 1.846 | 3877 |
| Grimelund | 0 | 2 | 0 | 0 | 1.550 | 3183 |

| **Neighborhood** (sub-districts) | **Number of restaurants** | **Number of grocery stores** | **Number of convenience stores** | **Number of fast food restaurants** | **Area** (km^2^) | **Population density** (population per km^2^) |
| --- | --- | --- | --- | --- | --- | --- |
| Grorud | 2 | 3 | 2 | 5 | 1.549 | 2653 |
| Grünerløkka vest | 28 | 4 | 4 | 7 | 0.560 | 15095 |
| Grünerløkka øst | 28 | 5 | 4 | 12 | 0.260 | 20988 |
| Grønland | 26 | 8 | 7 | 12 | 0.387 | 25214 |
| Hammersborg | 71 | 12 | 6 | 23 | 0.665 | 12370 |
| Hasle | 1 | 5 | 0 | 1 | 0.850 | 7218 |
| Haugenstua | 1 | 2 | 0 | 1 | 0.770 | 5096 |
| Hellerudtoppen | 0 | 1 | 0 | 0 | 0.759 | 4754 |
| Holmen | 0 | 4 | 1 | 0 | 1.542 | 3895 |
| Holmenkollen | 1 | 0 | 0 | 0 | 3.824 | 2379 |
| Holmlia Nord | 0 | 2 | 0 | 2 | 3.953 | 1499 |
| Holmlia Syd | 3 | 4 | 1 | 0 | 1.719 | 3478 |
| Homansbyen | 30 | 8 | 3 | 12 | 0.553 | 18964 |
| Hovseter | 0 | 5 | 1 | 1 | 2.043 | 3562 |
| Høybråten | 0 | 3 | 0 | 0 | 2.341 | 3287 |
| Ila | 7 | 2 | 2 | 4 | 0.640 | 14116 |
| Iladalen | 6 | 5 | 1 | 7 | 0.524 | 16076 |
| Kampen | 6 | 2 | 4 | 3 | 0.269 | 15160 |
| Kjelsås | 0 | 3 | 1 | 1 | 1.360 | 3914 |
| Korsvoll | 0 | 3 | 1 | 0 | 1.420 | 3871 |
| Kværnerbyen | 4 | 4 | 0 | 1 | 1.070 | 9018 |
| Lambertseter | 4 | 8 | 5 | 4 | 2.002 | 5303 |
| Lilleaker | 6 | 4 | 1 | 2 | 1.385 | 3996 |
| Lindeberg | 1 | 3 | 2 | 2 | 1.113 | 7109 |
| Lindern | 2 | 4 | 1 | 1 | 1.504 | 4521 |
| Linderud | 2 | 5 | 2 | 6 | 1.295 | 5454 |

| **Neighborhood** (sub-districts) | **Number of restaurants** | **Number of grocery stores** | **Number of convenience stores** | **Number of fast food outlets** | **Area** (km^2^) | **Population density** (population per km^2^) |
| --- | --- | --- | --- | --- | --- | --- |
| Ljan | 0 | 1 | 0 | 0 | 4.877 | 1187 |
| Løren | 7 | 7 | 1 | 4 | 1.060 | 10164 |
| Majorstuen nord | 13 | 6 | 7 | 5 | 0.424 | 12219 |
| Majorstuen syd | 17 | 6 | 2 | 7 | 0.352 | 25395 |
| Manglerud | 2 | 8 | 4 | 6 | 2.594 | 4236 |
| Montebello-Hoff | 1 | 5 | 2 | 0 | 2.467 | 3360 |
| Mortensrud | 1 | 2 | 1 | 2 | 3.225 | 1649 |
| Munkerud | 4 | 4 | 0 | 1 | 2.308 | 3869 |
| Myrer | 0 | 2 | 0 | 1 | 1.006 | 5779 |
| Nedre Tøyen | 4 | 3 | 0 | 3 | 0.197 | 26817 |
| Nordberg | 1 | 4 | 2 | 5 | 3.293 | 2858 |
| Nordstrand | 3 | 2 | 0 | 2 | 3.265 | 2444 |
| Nordtvet | 3 | 4 | 3 | 6 | 1.462 | 3716 |
| Oppsal | 2 | 5 | 1 | 2 | 1.661 | 6735 |
| Prinsdal | 1 | 3 | 0 | 6 | 3.431 | 1697 |
| Refstad | 2 | 3 | 0 | 1 | 1.464 | 5250 |
| Rodeløkka | 12 | 9 | 2 | 4 | 0.459 | 20815 |
| Rommen | 0 | 3 | 0 | 2 | 1.354 | 2649 |
| Romsås | 0 | 3 | 2 | 2 | 2.026 | 3326 |
| Røa | 6 | 9 | 0 | 1 | 3.408 | 3137 |
| Rødtvet | 0 | 1 | 1 | 0 | 0.694 | 5036 |
| Sagene | 5 | 4 | 1 | 2 | 0.345 | 17881 |
| Sandaker | 9 | 10 | 1 | 8 | 0.694 | 18709 |
| Sentrum | 123 | 16 | 44 | 44 | 2.653 | 535 |
| Simensbråten | 2 | 4 | 1 | 2 | 2.492 | 3756 |
| Sinsen | 7 | 6 | 0 | 4 | 0.488 | 19309 |
| Skillebekk | 46 | 7 | 3 | 8 | 1.990 | 4679 |

| **Neighborhood** (sub-districts) | **Number of restaurants** | **Number of grocery stores** | **Number of convenience stores** | **Number of fast food outlets** | **Area** (km^2^) | **Population density** (population per km^2^) |
| --- | --- | --- | --- | --- | --- | --- |
| Skullerud | 2 | 4 | 2 | 1 | 1.640 | 4559 |
| Skøyen | 5 | 4 | 2 | 1 | 2.375 | 3592 |
| Slemdal | 2 | 0 | 0 | 0 | 2.620 | 2814 |
| Sofienberg | 11 | 5 | 1 | 4 | 0.879 | 10430 |
| Stovner | 0 | 1 | 1 | 0 | 1.588 | 3622 |
| Teisen | 0 | 3 | 0 | 1 | 1.679 | 3426 |
| Torshov | 8 | 6 | 2 | 5 | 0.853 | 12399 |
| Trosterud | 0 | 2 | 4 | 2 | 3.227 | 2778 |
| Tveita | 0 | 5 | 4 | 3 | 1.812 | 3134 |
| Tåsen | 3 | 2 | 3 | 9 | 1.674 | 4692 |
| Ullern | 0 | 1 | 3 | 2 | 1.930 | 3086 |
| Ullernåsen | 0 | 2 | 0 | 0 | 1.614 | 4079 |
| Ullevål hageby | 2 | 4 | 0 | 2 | 2.050 | 3328 |
| Ulven | 2 | 2 | 1 | 2 | 2.435 | 1600 |
| Uranienborg | 19 | 4 | 2 | 5 | 0.548 | 13922 |
| Veitvet | 2 | 3 | 1 | 0 | 1.137 | 5650 |
| Vestli | 2 | 1 | 2 | 1 | 1.766 | 4027 |
| Vinderen | 2 | 2 | 1 | 2 | 1.580 | 3278 |
| Vålerenga | 6 | 3 | 1 | 4 | 0.532 | 11049 |
| Årvoll | 0 | 2 | 1 | 2 | 1.592 | 5611 |

Table S4. Characteristics of the physical activity environment in Oslo neighborhoods

| **Neighborhood** (sub-districts) | **Number of green spaces** | **Total area of green spaces** (m^2^x10^3^) | **Number of indoor facilities** | **Number of small outdoor facilities** | **Number of large outdoor facilities** | **Number of public transportation stops^1^** | **Area** (km^2^) | | | **Population density** (population per km^2^) |  |
| --- | --- | --- | --- | --- | --- | --- | --- | --- | --- | --- | --- |
| Abildsø | 17 | 48.0 | 1 | 13 | 11 | 8 | | 1.668 | 2294 | | |
| Ammerud | 15 | 15.4 | 4 | 11 | 0 | 11 | | 1.315 | 5839 | | |
| Bekkelaget | 3 | 160.1 | 6 | 25 | 28 | 25 | | 12.175 | 814 | | |
| Bislett | 7 | 1.3 | 4 | 6 | 1 | 13 | | 0.428 | 22348 | | |
| Bispevika | 9 | 16.1 | 1 | 0 | 0 | 12 | | 10.240 | 356 | | |
| Bjølsen | 9 | 8.5 | 4 | 23 | 12 | 4 | | 0.693 | 10850 | | |
| Bjørndal | 11 | 16.9 | 3 | 32 | 7 | 22 | | 6.289 | 1437 | | |
| Bjørnerud | 14 | 32.7 | 3 | 22 | 3 | 11 | | 1.489 | 4708 | | |
| Bygdøy | 12 | 13.4 | 5 | 14 | 4 | 12 | | 8.385 | 450 | | |
| Bøler | 12 | 30.3 | 6 | 6 | 1 | 9 | | 1.551 | 5444 | | |
| Disen | 9 | 5.1 | 0 | 6 | 5 | 10 | | 1.040 | 4572 | | |
| Dælenenga | 3 | 1.4 | 5 | 4 | 1 | 5 | | 0.196 | 22918 | | |
| Ellingsrud | 23 | 30.8 | 4 | 14 | 4 | 5 | | 1.594 | 4698 | | |
| Enerhaugen | 15 | 5.4 | 0 | 3 | 1 | 7 | | 0.398 | 19018 | | |
| Ensjø | 17 | 3.6 | 3 | 8 | 6 | 10 | | 1.250 | 7724 | | |
| Etterstad | 12 | 13.2 | 2 | 5 | 0 | 9 | | 0.931 | 4685 | | |
| Fagerborg | 4 | 6.6 | 0 | 9 | 0 | 6 | | 0.339 | 15454 | | |
| Fossum | 17 | 9.9 | 1 | 8 | 1 | 3 | | 0.431 | 12084 | | |
| Frogner | 14 | 7.5 | 0 | 2 | 0 | 9 | | 1.158 | 5983 | | |
| Frognerparken | 4 | 14.9 | 0 | 25 | 1 | 8 | | 0.660 | 10256 | | |
| Furuset | 43 | 42.9 | 7 | 20 | 8 | 16 | | 3.510 | 2898 | | |
| Godlia | 19 | 55.6 | 1 | 10 | 0 | 19 | | 2.717 | 3265 | | |
| Grefsen | 13 | 7.5 | 7 | 14 | 2 | 24 | | 1.846 | 3877 | | |
| Grimelund | 10 | 9.8 | 0 | 3 | 0 | 11 | | 1.550 | 3183 | | |

| **Neighborhood** (sub-districts) | **Number of green spaces** | **Total area of green spaces** (m^2^x10^3^) | **Number of indoor facilities** | **Number of small outdoor facilities** | **Number of large outdoor facilities** | **Number of public transportation stops^1^** | **Area** (km^2^) | **Population density** (population per km^2^) |
| --- | --- | --- | --- | --- | --- | --- | --- | --- |
| Grorud | 14 | 19.8 | 2 | 7 | 3 | 14 | 1.549 | 2653 |
| Grünerløkka vest | 17 | 10.4 | 8 | 9 | 0 | 5 | 0.560 | 15095 |
| Grünerløkka øst | 5 | 4.5 | 0 | 0 | 0 | 12 | 0.260 | 20988 |
| Grønland | 4 | 2.6 | 4 | 10 | 0 | 11 | 0.387 | 25214 |
| Hammersborg | 9 | 1.8 | 5 | 10 | 0 | 15 | 0.665 | 12370 |
| Hasle | 4 | 7.8 | 6 | 7 | 11 | 7 | 0.850 | 7218 |
| Haugenstua | 29 | 12.8 | 1 | 18 | 2 | 9 | 0.770 | 5096 |
| Hellerudtoppen | 2 | 3.8 | 0 | 2 | 0 | 12 | 0.759 | 4754 |
| Holmen | 13 | 7.0 | 1 | 18 | 2 | 13 | 1.542 | 3895 |
| Holmenkollen | 23 | 24.5 | 2 | 11 | 0 | 22 | 3.824 | 2379 |
| Holmlia Nord | 19 | 71.2 | 2 | 18 | 4 | 11 | 3.953 | 1499 |
| Holmlia Syd | 21 | 50.3 | 5 | 15 | 1 | 15 | 1.719 | 3478 |
| Homansbyen | 7 | 0.6 | 2 | 3 | 0 | 16 | 0.553 | 18964 |
| Hovseter | 20 | 19.4 | 6 | 18 | 3 | 14 | 2.043 | 3562 |
| Høybråten | 35 | 20.4 | 3 | 14 | 6 | 15 | 2.341 | 3287 |
| Ila | 15 | 12.4 | 3 | 8 | 0 | 8 | 0.640 | 14116 |
| Iladalen | 8 | 6.9 | 3 | 4 | 1 | 7 | 0.524 | 16076 |
| Kampen | 5 | 4.2 | 2 | 5 | 0 | 4 | 0.269 | 15160 |
| Kjelsås | 10 | 22.3 | 4 | 8 | 2 | 16 | 1.360 | 3914 |
| Korsvoll | 31 | 17.4 | 1 | 13 | 3 | 10 | 1.420 | 3871 |
| Kværnerbyen | 13 | 9.2 | 0 | 13 | 0 | 12 | 1.070 | 9018 |
| Lambertseter | 19 | 54.7 | 6 | 16 | 3 | 20 | 2.002 | 5303 |
| Lilleaker | 9 | 8.2 | 2 | 3 | 0 | 10 | 1.385 | 3996 |
| Lindeberg | 16 | 14.8 | 3 | 20 | 4 | 8 | 1.113 | 7109 |
| Lindern | 16 | 11.5 | 1 | 8 | 1 | 15 | 1.504 | 4521 |
| Linderud | 11 | 13.1 | 1 | 14 | 1 | 16 | 1.295 | 5454 |

| **Neighborhood** (sub-districts) | **Number of green spaces** | **Total area of green spaces** (m^2^x10^3^) | **Number of indoor facilities** | **Number of small outdoor facilities** | **Number of large outdoor facilities** | **Number of public transportation stops^1^** | **Area** (km^2^) | **Population density** (population per km^2^) |
| --- | --- | --- | --- | --- | --- | --- | --- | --- |
| Ljan | 25 | 14.3 | 4 | 13 | 1 | 14 | 4.877 | 1187 |
| Løren | 1 | 2.2 | 3 | 12 | 1 | 6 | 1.060 | 10164 |
| Majorstuen nord | 2 | 0.0 | 2 | 1 | 2 | 9 | 0.424 | 12219 |
| Majorstuen syd | 4 | 0.3 | 0 | 0 | 0 | 7 | 0.352 | 25395 |
| Manglerud | 22 | 51.8 | 6 | 12 | 2 | 18 | 2.594 | 4236 |
| Montebello-Hoff | 33 | 13.3 | 5 | 6 | 1 | 18 | 2.467 | 3360 |
| Mortensrud | 23 | 42.0 | 1 | 5 | 4 | 18 | 3.225 | 1649 |
| Munkerud | 13 | 14.1 | 4 | 11 | 4 | 18 | 2.308 | 3869 |
| Myrer | 4 | 1.9 | 1 | 11 | 3 | 10 | 1.006 | 5779 |
| Nedre Tøyen | 7 | 2.3 | 1 | 7 | 0 | 5 | 0.197 | 26817 |
| Nordberg | 34 | 23.9 | 6 | 31 | 7 | 27 | 3.293 | 2858 |
| Nordstrand | 20 | 14.0 | 3 | 18 | 0 | 20 | 3.265 | 2444 |
| Nordtvet | 6 | 7.8 | 2 | 4 | 2 | 15 | 1.462 | 3716 |
| Oppsal | 18 | 17.4 | 5 | 14 | 5 | 16 | 1.661 | 6735 |
| Prinsdal | 10 | 3.1 | 0 | 1 | 1 | 15 | 3.431 | 1697 |
| Refstad | 6 | 2.1 | 2 | 12 | 3 | 8 | 1.464 | 5250 |
| Rodeløkka | 4 | 1.8 | 0 | 4 | 0 | 7 | 0.459 | 20815 |
| Rommen | 23 | 15.7 | 0 | 4 | 6 | 6 | 1.354 | 2649 |
| Romsås | 16 | 23.1 | 4 | 11 | 3 | 17 | 2.026 | 3326 |
| Røa | 36 | 27.5 | 1 | 14 | 3 | 17 | 3.408 | 3137 |
| Rødtvet | 8 | 7.8 | 2 | 8 | 2 | 3 | 0.694 | 5036 |
| Sagene | 9 | 6.3 | 0 | 3 | 0 | 5 | 0.345 | 17881 |
| Sandaker | 9 | 3.0 | 0 | 2 | 1 | 12 | 0.694 | 18709 |
| Sentrum | 34 | 36.6 | 0 | 1 | 0 | 50 | 2.653 | 535 |
| Simensbråten | 21 | 25.2 | 2 | 7 | 6 | 17 | 2.492 | 3756 |
| Sinsen | 3 | 0.9 | 0 | 2 | 0 | 9 | 0.488 | 19309 |
| Skillebekk | 18 | 4.2 | 5 | 6 | 0 | 15 | 1.990 | 4679 |

| **Neighborhood** (sub-districts) | **Number of green spaces** | **Total area of green spaces** (m^2^x10^3^) | **Number of indoor facilities** | **Number of small outdoor facilities** | **Number of large outdoor facilities** | **Number of public transportation stops^1^** | **Area** (km^2^) | **Population density** (population per km^2^) |
| --- | --- | --- | --- | --- | --- | --- | --- | --- |
| Skullerud | 19 | 35.6 | 1 | 3 | 5 | 12 | 1.640 | 4559 |
| Skøyen | 19 | 45.5 | 4 | 26 | 2 | 11 | 2.375 | 3592 |
| Slemdal | 14 | 8.7 | 6 | 28 | 5 | 11 | 2.620 | 2814 |
| Sofienberg | 13 | 34.0 | 5 | 15 | 1 | 14 | 0.879 | 10430 |
| Stovner | 36 | 11.3 | 2 | 11 | 0 | 11 | 1.588 | 3622 |
| Teisen | 9 | 8.0 | 1 | 4 | 1 | 17 | 1.679 | 3426 |
| Torshov | 17 | 23.0 | 1 | 19 | 1 | 18 | 0.853 | 12399 |
| Trosterud | 23 | 11.6 | 2 | 15 | 1 | 24 | 3.227 | 2778 |
| Tveita | 23 | 32.7 | 1 | 9 | 3 | 18 | 1.812 | 3134 |
| Tåsen | 27 | 20.9 | 0 | 5 | 0 | 17 | 1.674 | 4692 |
| Ullern | 8 | 7.1 | 2 | 11 | 0 | 11 | 1.930 | 3086 |
| Ullernåsen | 21 | 30.2 | 3 | 12 | 4 | 7 | 1.614 | 4079 |
| Ullevål hageby | 20 | 9.7 | 10 | 22 | 1 | 18 | 2.050 | 3328 |
| Ulven | 14 | 5.2 | 2 | 11 | 0 | 22 | 2.435 | 1600 |
| Uranienborg | 10 | 2.4 | 4 | 4 | 0 | 16 | 0.548 | 13922 |
| Veitvet | 7 | 2.9 | 4 | 13 | 2 | 7 | 1.137 | 5650 |
| Vestli | 43 | 35.7 | 4 | 23 | 2 | 12 | 1.766 | 4027 |
| Vinderen | 5 | 2.9 | 3 | 3 | 0 | 19 | 1.580 | 3278 |
| Vålerenga | 7 | 5.3 | 6 | 8 | 2 | 5 | 0.532 | 11049 |
| Årvoll | 16 | 8.5 | 2 | 12 | 5 | 15 | 1.592 | 5611 |

^1^Number of stops including bus, train, tram, and metro
